# Abnormal Glycosylation of Cancer Stem Cells and Targeting Strategies

**DOI:** 10.3389/fonc.2021.649338

**Published:** 2021-04-06

**Authors:** Thahomina Khan, Horacio Cabral

**Affiliations:** Department of Bioengineering, Graduate School of Engineering, The University of Tokyo, Tokyo, Japan

**Keywords:** sialic acid (N-acetyl neuraminic acid), phenylboronic acid chemistry, sialyltransferase (ST), CSC markers, stemness signaling pathway

## Abstract

Cancer stem cell (CSCs) are deemed as one of the main reasons of tumor relapse due to their resistance to standard therapies. Numerous intracellular signaling pathways along with extracellular features are crucial in regulating CSCs properties, such as heterogeneity, plasticity and differentiation. Aberrant glycosylation of these cellular signaling pathways and markers of CSCs have been directly correlated with maintaining survival, self-renewal and extravasation properties. In this review, we highlight the importance of glycosylation in promoting stemness character of CSCs, and present strategies for targeting abnormal glycosylation to eliminate the resistant CSC population.

## Introduction

The emergence of drug resistance and relapse of tumors have maintained the rate of cancer related deaths despite the advances in cancer treatment. Recent studies have associated the formation of metastasis, resistance to therapy, and eventual tumor relapse with the presence of a tumorigenic subpopulation of cancer cells showing stem-like features within the cellular heterogeneity of tumors (1). These cancer stem-like cells (CSCs) are deemed as one of the main reasons of tumor relapse (2). The presence of CSCs has been reported in different type of cancers, *e.g.* head and neck cancer (3), liver cancer (4), breast cancer (5), brain cancer (6) and melanoma (7). While the origin of CSCs is still not clear, the source may be a result of epithelial to mesenchymal transition (EMT), which allows differentiated and non-stem cells to develop characters like CSCs (8), or the alteration of non-malignant stem cells to CSCs due to oncogenic somatic mutation (9). In recent years several upregulated markers of CSCs have been identified, such as epithelial cell-adhesion molecule (EpCAM), CD44, CD133, CD24 and aldehyde dehydrogenase (ALDH) (1). These markers are being used alone or in combination in different tumors for isolating CSCs with drug resistance and self-renewal ability (10). Moreover, several cellular signaling pathways, such as Notch, hedgehog, Wnt/β-catenin, Akt, NF-_K_B, JAK-STAT and PPAR, which are also present in healthy stem cells, are being studied extensively due to their effect in self-renewal, metastasis and immune evasion (11). However, it is important to note that CSCs are highly heterogeneous, showing variability between tumors and even within the same tumor tissue (12). Thus, dealing with common targets shared by CSCs could allow developing far reaching therapies capable of treating a wide range of tumors.

Altered glycosylation ensuing modifications in proteins during or after translation (13) is a trademark of almost all type of cancers regardless of the origin and stage (14), and it is a common feature of CSC population markers and signaling pathways (15). The glycan chains on cancer cells regulate a range of pathological processes, including cell-cell interaction, cell adhesion, receptor activation and signal transduction (14). The main terminal sugar of the cell surface glycan chain is neuraminic acid (sialic acid; SA), which is being studied extensively in tumor biology (16). The overexpression of terminal glycan SA has also been linked to cancer malignancy and metastasis (16, 17). Moreover, hypersialylation in cancer cells promotes cell migration (18) and apoptosis resistance (19), which lead to tumor growth and poor prognosis (16). CSCs may be further distinguished from differentiated cancer cells by the expression of carbohydrate antigens (20, 21), and the N-glycosylation of glycoproteins of CSCs has been related to drug resistance mechanisms (22). Besides, abnormal glycosylation has been shown to regulate signaling pathways and markers involved in conservation of CSCs, such as EpCAM glycosylation on cell adhesion (23) and EMT in breast cancer, or N-glycosylation on MAPK and PI3/Akt pathway (24). As the carbohydrate antigens of glycan chains are primarily located on the plasma membrane of cells, they are regarded as outstanding biomarkers for detecting altered phases of cellular differentiation (21) and could provide opportunities for generating targeted therapies.

Herein, we review the altered glycosylation of cellular pathways and markers involved in CSCs maintenance and resistance mechanisms. The possibilities to use the glycosylation of both cancer cells and CSCs as targets for tumor treatment are also presented. Finally, we discuss the future perspectives in this bourgeoning field.

## CSC and Altered Glycosylation

**Figure 1:**
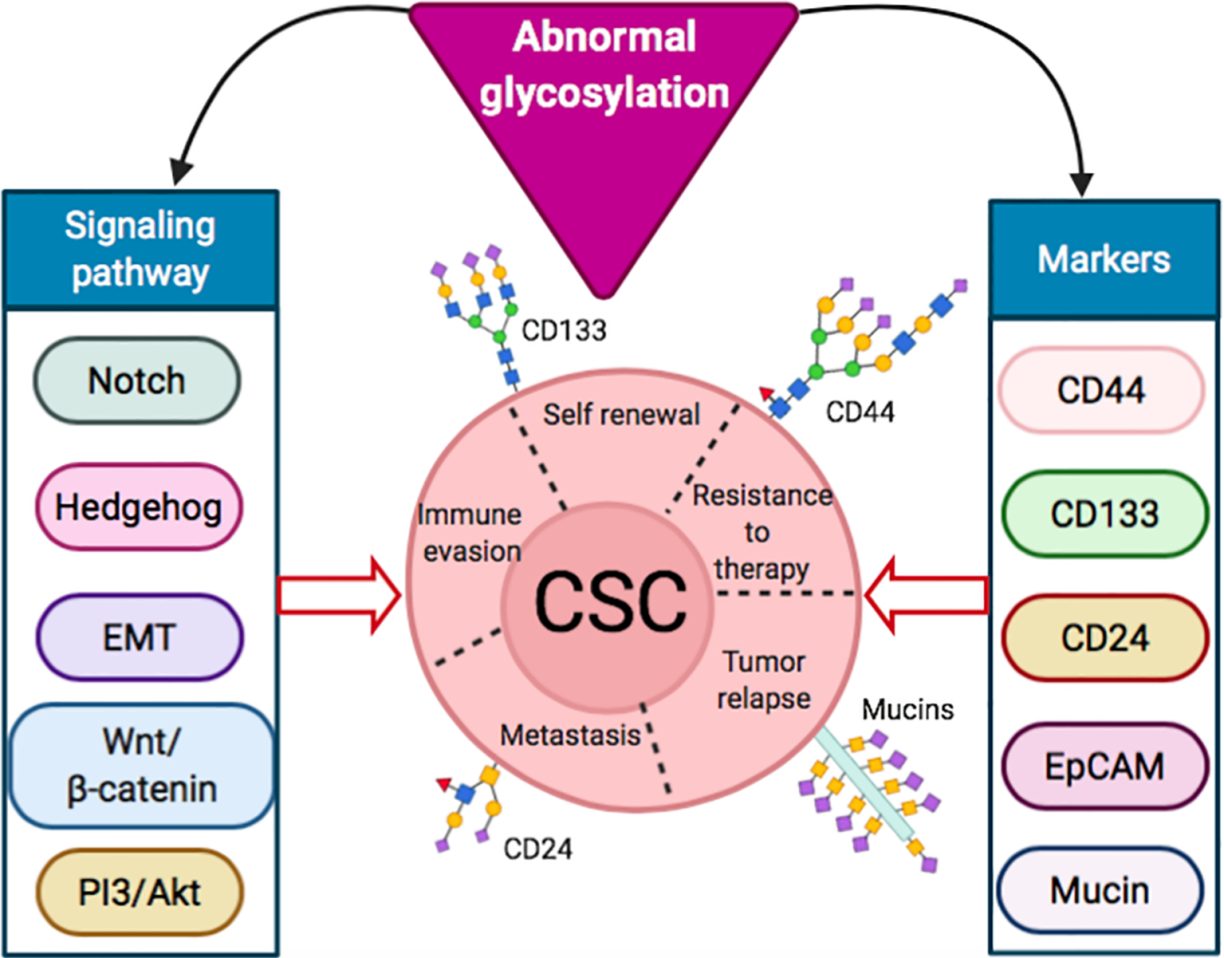
Abnormal glycosylation in signaling pathways and markers inducing stemness properties of CSCs.

The importance of altered glycosylation of tumor in disease progression has been well documented (14). In the following sections, we focused on the aberrant glycosylation in CSC markers, such as CD44, CD133 and CD24, along with different cellular signaling and pathways, like Notch (25), hedgehog (26), Wnt/β-catenin (27) and Akt (28), in maintaining CSC properties (11, 15) (**Figure 1**). A summary of the role of signaling pathways and markers in normal cells and CSCs, along with their altered glycosylation reported so far, is presented in **Table 1**.

**Table 1:** Glycan abnormalities in CSC signaling pathways and markers.

Pathways and markers in CSC		Role in normal cells	Role in cancer stem cells	Glycan abnormalities
Signaling pathway	Notch	Cell-cell communication, proliferation, homeostasis and apoptosis (29, 30)	Renewal ability, Immune escape (29, 30)	Fucosyltransferase, Fringe family N-acetyl-glucosaminidyl-transferases, glycosyltransferase GnT-III (31, 32)
	Hedgehog	Proliferation, migration and differentiation of embryonic cells (26, 33)	Maintenance and regeneration (26, 33)	GALNT1 (34)
	EMT	Inhibition of cell-cell adhesion, modulation of polarity, downregulation of cytokeratin (35)	Increased plasticity, stemness and migration (9)	GALNT3, GALNT6, GALNT14 (36, 37)
	Wnt/β-catenin	Cell migration, polarity, neural patterning (38, 39)	Renewal, cell proliferation and differentiation (27, 40)	N-Acetylglucosaminyltransferase-V (GnT-V) ST6 N-Acetylgalactosaminide Alpha-2,6-Sialyltransferase 1 (ST6GALNAC1) (41, 42)
	PI3/Akt	Growth, proliferation, metabolism, motility, survival, and apoptosis (43)	Survival and proliferation (28)	ST6GALNAC1 (44)
Markers	CD44	Cellular adhesion, receptor for hyaluronic acid, release of cytokines (45, 46)	Self-renewal, tumor initiation and metastasis (3, 47, 48)	Altered N- and O- linked glycosylation, GALNT3 (49)
	CD133	Progenesis, neovascularization and hair follicle regeneration (50, 51)	Tumor initiation and drug resistance (52, 53)	Sialylation of N-glycan terminal *via* α2,3-site and STn (54, 55)
	CD24	Cell-cell and cell-matrix interactions (56, 57)	Cell adhesion and metastasis (58–60)	Siglec 10 and sialylLewisx (58, 61)
	EpCAM	Cell adhesion, signaling, proliferation, differentiation (62, 63)	Cell migration, upregulation of proto oncogenic activities and chemoresistance (64, 65)	Glycosylation at Asn198 (63)
	Mucin	Protection, repair, transmission of cellular signals (66)	Resistance to apoptosis and chemotherapy (67, 68)	GALNT-6, Mucin-O-glycosylation of α2M (69)

### Glycosylation of Signaling Pathways

The involvement of glycosylation in dysregulation of signaling pathways is gaining much attention due to its key roles in ligand binding, signaling of receptors, and transport control inside the cells. Besides, glycosylation affects receptor turnover on cell membrane receptors as glycans binds to galectins to form a lattice. In addition, the binding of glycosylated proteins with gangliosides can modulate the intracellular communication of receptors.

For Notch signaling, which supports the renewal ability of CSCs and plays significant roles in the immune escape of and invasion and extravasation of cancer cells, the response are mediated by 4 Notch transmembrane receptor and 5 ligands (3 Delta-like and 2 Jagged) (25). The affinity of these Notch receptors to ligands is regulated by the glycosylation of receptors (31). Specially, through insertion of fucose-N-acetylglucosamine by fucosyltransferase and Fringe family N-acetyl-glucosaminidyl-transferases (31). Various glycosyltransferases take part in the glycosylation of Notch in various tumor types (70). Regulation of Notch signaling is associated with tumor stemness and metastasis progression (25, 33).

Hedgehog (Hh) signaling is crucial for maintaining and regenerating CSCs (26, 33). The Hh signaling is a complex pathway regulated by different receptors, such as the transmembrane protein receptor PTCH and various extracellular Hh ligands and sonic Hh ligand (SHH) and the transmembrane protein SMO (71). GALNT1 a glycotransferase have been identified by researchers to play a pivotal role in SHH instigation in CSC of bladder cancer (34). GALNT1 regulated O-linked glycosylation foster activation of SHH signaling, which is vital for self-regeneration of CSCs (34).

Glycosylation also maintains tumor epithelial plasticity, including EMT driven by hexosamine biosynthetic pathway (HBP) and O-GlcNAcylation leading to chemoresistance of cancer cells (72). Increased expression of GALNT3 or GALNT6 correlates with the O-glycosylation-mediated EMT in epithelial cells of human prostate (36). Moreover, GALNT14 increase EMT genes, migration and invasion of breast cancer cells (37). EMT in breast cancer is also affected by the N-glycosylation of EpCAM *via* multiple cell signaling pathways (24). N-glycosylation promoted EMT also stabilizes and upregulates programmed death-ligand 1 (PD-L1), contributing to the immune escape of CSCs (73, 74).

N-acetylglucosaminyltransferase-V (GnT-V) and ST6 N-Acetylgalactosaminide alpha-2,6-Sialyltransferase 1 (ST6GALNAC1) facilitated glycosylation have been reported to control stemness of colon cancer cells through WNT signaling and Akt pathway respectively (41, 42, 44). GnT-V induces the N-glycosylation of receptors present on Wnt proteins, which alters normal Wnt/β-catenin signaling pathway (41). Such alteration causes distorted development of adenoma through regulation of CSC in colorectal cancer (41). The role of PI3K-Akt pathway in supporting proliferation and stemness characteristics of CSC in breast, brain and colorectal cancer cells have been studied thoroughly (75–77). ST6GALNAC1 may support conservation of CSCs through activation Akt pathway by facilitating recruitment of sialyl-Thomsen-nouveau (STn) antigen, which interacts with galectin-3 (44). STn is the consequence of premature sialylation of Tn antigen due to upregulation of ST6GALNAC1 and disturbance of elongation steps (78). ST6GALNAC1 facilitates recruitment of STn through addition of SA on GALNAC and backbone of protein. The STn antigen inside cells activates galectin-3 mediating phosphorylation of Akt, which decreases GSK3β and promotes protein transcription by β-catenin. Phosphorylated Akt also fosters S6 phosphorylation by mTORC1 leading to augmented protein synthesis, which triggers stemness through stimulated transcription (44). Expression of ST6 beta-galactoside alpha-2,6-sialyltransferase 1(ST6GAL1), which adds alpha2-6 lined sialic acid to glycosylated protein, is also upregulated in cancer malignancies specially in CSCs (79, 80). ST6GAL1 expression corelated with other CSC markers in colorectal carcinoma samples from patient and induced chemoresistance (81). In ovarian cancer, ST6GAL1 expression is regulated by SOX2, a well-known stem cell transcription factor (82).

### Glycosylation of CSC Markers

Most CSC markers are glycoproteins, such as CD44, CD133 or CD24, expressing various glycan moieties on cell surface (15). These glycans are fundamental in many biological processes. For example, the terminal glycan SA is involved in regulating pluripotency of embryonic stem cells (20). Indeed, SA-cleaving promotes differentiation (20). Glycosylation of CSC markers regulates many functions of CSCs, including cell adhesion, extravasation, evasion of immune cells and apoptosis, self-regeneration and preservation of pluripotency (15).

CD44 is a transmembrane glycoprotein with the ability to activate EGFR and ErbB‐2 by binding to hyaluronic acid (HA) on extracellular matrix. Such activation has been linked to increased cell differentiation and relocation (45). While CD44 can be found on healthy cells, such as leukocytes, mesenchymal cells, endothelial and parenchymal liver cells (46), it is also a major player in different types of cancers, like head and neck, skin, lung, breast, prostate, pancreatic and liver cancer (47, 48). CD44 is hailed as a broad-ranging biomarker for CSCs. Moreover, isoforms of CD44 (CD44v), which are rarer than standard CD44, have also been linked with CSC subpopulations (3). Both the standard and variants of CD44 are reported to have altered N- and O- linked glycosylation (83). Such differences in glycosylation are associated with altered molecular weights (84, 85). Five probable N-linked glycosylation locations on CD44 can initiate cell binding through HA (83). Moreover, glycosylation of CD44 could control HA adhesion, as demonstrated in ovarian cancer (86), amplifying or inhibiting attachment of CD44 to HA. For example, improved binding has been reported by O-linked glycans (N-deglycosylated), N-linked N-acetylglucosamine and N-acetylgalactosamine recruitment in non-N-linked glycans on CD44. On the contrary, α2,3- linked SA on N-linked glycans have opposite effect and hinders such binding (87). The expression of SA in particularly high on CD44 positive CSCs (44, 88). In addition, the distorted glycosylation of CD44v and their expression have been linked with the aggressiveness of cancer in human patients (89).

Another extensively used CSC marker is CD133 or prominin-1 (28), which is associated with the Wnt signaling (90), and the PI3K/Akt pathway (91) in CSCs. CSCs identified with CD133+ markers in patients with ovarian cancer correspond to poor survival (92). CD133 is encoded by PROM1 gene composed of 8 N-linked glycosylation sites (93), and the glycosylation plays important roles in maintaining CSC properties (94, 95). In fact, glycosylation of CD133 has been suggested as a secondary indicator of CSCs (96–98). In particular, sialylation of CD133 N-glycan terminal *via* α2,3-site was found in the CSC of glioma (54), which was augmented in hypoxic conditions, correlating with the migration and survival of brain CSCs (99). Moreover, CD133 could be co-expressed with STn, as found in a subset of ovarian cancer cells (55).

CD24, which was originally identified as a B cell differentiation marker (100), has been indicated as a CSC marker in several tumors, such as ovarian (101), colorectal (102), bladder (103) and urothelial (104) cancers. CD24 has been directly correlated with disease progression and metastasis (59, 60, 105, 106). CD24 is a glycoprotein having both N- and O-linked type glycosylation and linked to glycosylphosphatidylinositol (61). CD24-expressing cells can evade the immune system by binding to Siglec-10 (a SA binding molecule) present on macrophages (61). Moreover, binding of CD24 to immune cells through a sialylation dependent way initiates a cascade facilitated by SHP-1/SHP-2 leading to inactivation of immune cells (107). Apart from Siglecs, CD24 also acts as a ligand for P-selectin, an adhesion receptor expressed on platelets and endothelial cells (108). Thus, binding of cancer cells to P-selectin *via* CD24 may be crucial for inducing tumor metastasis (108). Moreover, sialylLewis^x^ (sialylLe^x^), a SA-bearing glycan, controls CD24 facilitated rolling of cancer cells on P-selectin and extravasation during metastasis (58).

EpCAM is expressed on different types of epithelial cells, stem cells, cancer cells and CSCs (63). EpCAM is a cell surface glycoprotein with 3 N-glycosylation sites (109), with glycosylation at Asn198 being the most important for stability (63). EpCAM+, CD44+, CD24- CSCs were 10-fold more likely to induce tumors compared to EpCAM-, CD44+, CD24- CSCs in breast cancer (110). In hepatocellular carcinoma, EpCAM+ CD133+ cells demonstrated elevated colony formation ability, expression of stem cell related genes and chemoresistance compared to EpCAM- CD133+ cells (111). Moreover, inhibition of EpCAM in resistant head and neck cancer cells sensitize them to cisplatin (64). EpCAM in carcinoma tissues presented elevated levels of glycosylation compared to normal epithelia (112). Particularly, elevated glycosylation at Asn198 mediates EpCAM stability and surface retention in HEK293 cells (112). The deglycosylation of EpCAM repressed proliferation of breast cancer cells and fostered apoptosis (113). More recent research demonstrated the role of N-glycosylation of EpCAM in maintaining stemness property and EMT in hypoxic condition (114).

Mucins are heavily glycosylated extracellular proteins, which are mostly O-linked type, that have major impact on cell differentiation, adhesion and metastasis (67). For example, GALNT-6 elevated mucin-O-glycosylation of α2M, which activates downstream PI3/Akt signaling pathway, fosters metastasis of breast cancer (69). CD44+, CD24+ and CD133+ CSC populations of pancreatic cancer also express Mucin-1 (115) and Mucin 5AC also plays significant role in maintaining stemness properties of pancreatic CSC (116). Increased fraction of CD44+, CD24- CSC and Mucin-1 expression was also reported in breast cancer MCF-7 cells when exposed to tumor associated macrophages (117). Mucin-1 have also been identified as an important factor in effectiveness of colorectal CSC vaccine (118). In case of gastric cancer metastasis, expression of Mucin-1 was detected in peripheral blood and bone marrow signifying its importance (119). Abnormal expression of Mucin-4 has also been reported in many cancers (120), and correlated with metastasis (121) and chemoresistance (122). Downregulation of Mucin-4 sensitizes CSCs of pancreatic cells to gemcitabine (123). Mucin-4 also helps to maintain CSC population of ovarian cancer cells by stabilizing expression of Her2 (124). Mucin-16 (CA125) enhancement is present in CSCs of both pancreatic (125) and ovarian (126, 127) cancer. In ALDH+ CSCs of pancreatic cancer, MUC16 (carboxyl terminal fragment) facilitates augmentation of JAK2, which upregulates *LMO2* and *NANOG* genes to provide stemness properties (128).

## Glycosylation in the Resistance of CSCs to Conventional Treatments

Eliminating CSCs represents a major challenge, as CSCs present less reactive oxygen species (129, 130) and are resistant to radio- and chemotherapy (129, 130) by increased efflux pumps, DNA repair and scavenger agents (129, 130). CSCs show phenotypical heterogeneities both at inter- and intratumoral levels (131, 132), which are associated with genetic mutations and epigenetic changes, or differences in tumor microenvironment, e.g. cytokines and hypoxia (131, 132). The heterogeneity of CSCs represents a major challenge for targeted therapies. Moreover, CSCs could exist in specialized environments within tumors by forming niches that promote their survival (133, 134). CSCs can also adjust their niche and keep homeostatic processes, such as EMT and angiogenesis (133, 134). The CSC niche is dynamic, changing with tumor progression, and adapting to the treatments. Moreover, the niche has the ability to revert non-tumorigenic cancer cells into CSCs through EMT-related processes (8, 9, 135). This fluctuation between cancer cells and CSCs populations implies that targeting only CSCs may not be sufficient, as residual cancer cells may repopulate CSCs. Thus, it can be envisioned that for developing effective therapies, the elimination of both cancer cells and CSCs should be considered for achieving safe and robust long-term responses.

The functions of glycans present on CSC markers in inducing chemoresistance are not fully understood. Nonetheless, studies have indicated that glycosylation may play key roles in the resistance of CSCs. For example, O-GlcNAcylation of GNB2L1 facilitates chemoresistance of gastric cancer by regulating EMT (136). Furthermore, resistance to chemotherapeutic drug doxorubicin in the CD44+/CD24- CSC population of breast tumors was reversed by silencing expression of glucosylceramide synthase, which is involved in glycolipid biosynthesis (137). Also, in breast cancer, treatment with doxorubicin induces resistance through glycosylation of O-GlcNAc (138). Doxorubicin activates Akt pathway leading to upregulation of HBP and eventually O-GlcNAcylation, which promotes survival of cells through deterring apoptotic function of caspases and stimulating pro-survival factors like NF-_K_B and Akt (138). Elevated expression of Mucin-4 has also been reported in CD133+ CSCs in pancreatic cancer, which facilitated resistance to gemcitabine (123). Moreover, CD133+ CSC population isolated from patients malignant primary neoplasm was also more resistant to gemcitabine than CD133- cancer cells (52). Overexpression of Mucin-4 in two pancreatic cell lines Panc-1 and Mia-PaCa-2 was correlated with aggressiveness and resistance to gemcitabine compared to non Mucin-4 expressing Panc-1- and Mia-PaCa-pSectag C (123). In addition, the overexpression of Mucin-4/sialomucin complex (SMC) may protect cancer cells from the antibody Herceptin, as well as immune cells, by covering surface antigens, thus, hindering their availability (139–141). Therefore, therapies capable of dealing with the altered glycosylation of CSCs could allow developing strategies overcoming the resistance of CSC to conventional treatments.

## Targeting Altered Glycosylation in Cancer Cells and CSCs

Treatment approaches against abnormal glycosylation are emerging as appealing options for effective tumor therapy (142). Inhibition of glycans can decrease CSCs ability for maintaining stemness, thereby, decreasing tumor proliferation. For example, by siRNA-mediated silencing of GALNT1, which control O-linked glycosylation activation of SHH signaling in CD44+ CSCs in bladder cancer to mediate stemness, the tumor growth was suppressed in a similar extent to cyclopamine in an orthotopic mouse model of bladder cancer (34). Moreover, inhibiting glycosylation cannot only make changes in CSCs, but also in the tumor microenvironment. For example, targeting the SA-siglec interaction can convert the immunosuppressive tumor microenvironment into an immunoactive environment, as overexpression of SA has been deemed as a major mechanism for cancer cells to evade detection by immune cells (143). The sialoglycan-siglec glycol-immune checkpoint can be targeted in CSCs. T cells inside tumors, specially CD8+ T cells, present elevated amount of Siglecs, which engage with heavily sialylated CSCs (143, 144). Besides, cancer cells expressing CD24 can escape detection by the immune system by interacting with the inhibitory receptor SA-binding Ig-like lectin 10 (Siglec-10). Blocking the CD24-Siglec-10 binding by monoclonal antibodies improves mice survival outcome by increasing phagocytosis of CD24+ cells (61).

Targeting glycosylated CSC markers with specific antibodies is another approach to aim CSC precisely (145). Among them, CD44 targeting appear as a promising approach for enhancing antitumor efficacy. For example, KMP-1 antibody targeting CD44 can inhibit cell division, relocation and adhesion, allowing tumor growth suppression in a mouse model of bladder cancer (146). The anti-CD44 antibody RG7356 have shown also the possibility to activate the immune system by initiating phagocytosis through macrophage recruitment in breast cancer model (147). Antibodies, such as L2A5, can also be used for targeting tumor specific STn and short glycans expressing terminal α2–6 SA (78), and promote tumor inhibition (148, 149). As STn is co-expressed with CD133, anti-STn antibody drug conjugates could suppress tumor growth, effectively diminishing CSCs (55). Other antibodies, such as the anti-sialyl-di-Lewis^a^ antibody FG129, are being tested for targeting tumor-associated glycans toward the development of tumor-selective treatments and diagnosis modalities (150).

In most cases, the abnormal glycosylation in tumor leads to overexpression of SA at the terminal carbohydrate of the glycan chains, providing a useful target (14). Thus, efforts are being made to develop therapeutic and diagnostic approaches directed towards aberrant sialylation (151). These tactics includes blocking SA by using glycomimetics (152, 153) or targeting overexpressed SA in tumor to deliver therapeutic agents (154–157). Glycomimetic drugs, such as Uproleselan (GMI 1271) and Rivipansel (GMI 1070), which are both selectin (SA binding molecule) inhibitors, are being tested in clinical trials for treating acute myeloid leukemia (NCT03616470) (158, 159). Another molecule GMI 1359 which inhibits both E-selectin and CXCR4 have recently started Phase 1b clinical trial for metastatic breast cancer (NCT04197999) (160–162). Intratumoral injection of glycomimetic agents has shown enhanced antitumor responses, which are mediated by CD8+ T cell attack to cancer cells (152). However, the systemic application of such glycomimetic strategies blocking SA is difficult due to increased side effects.

**Figure 2:**
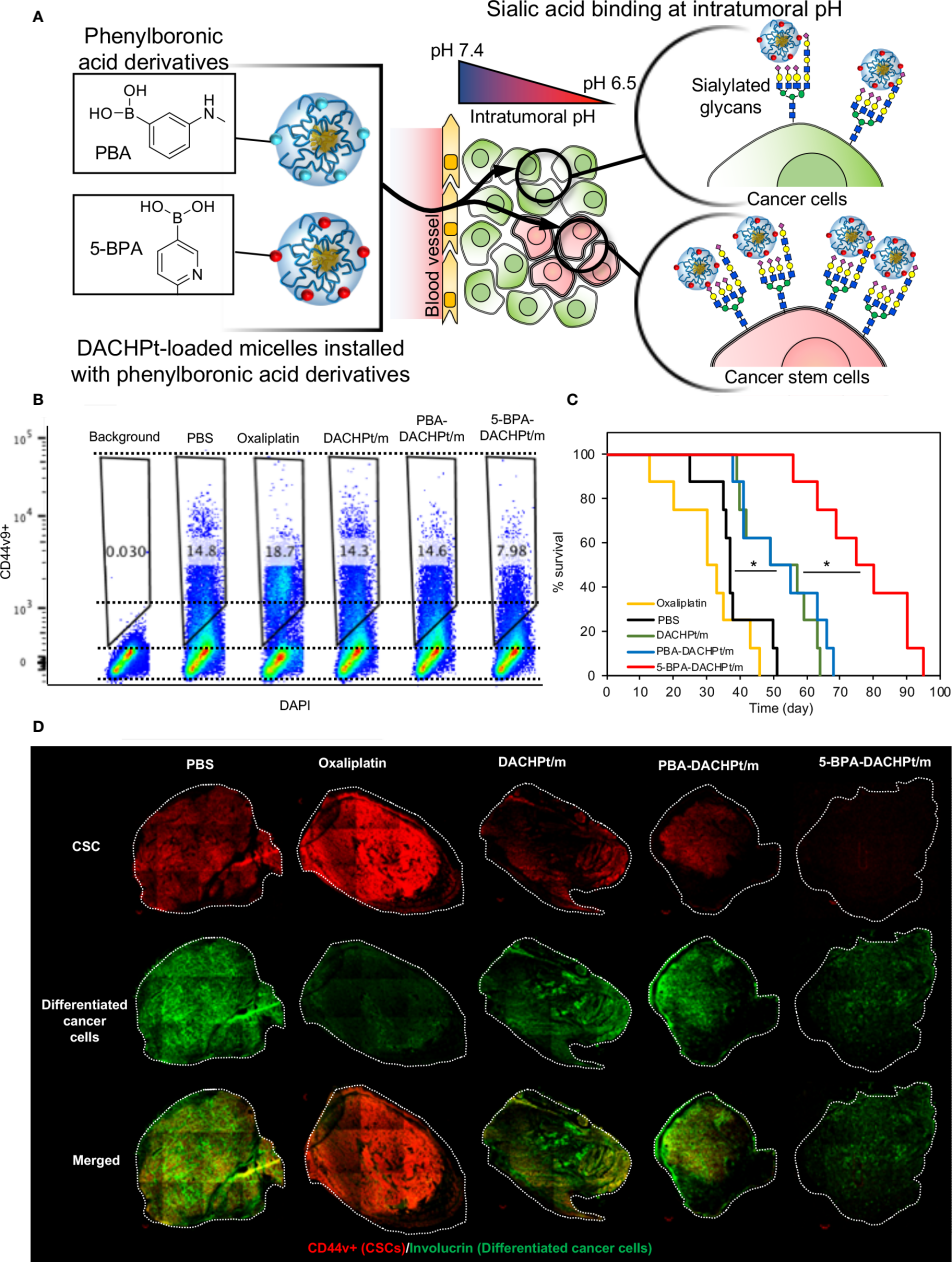
Targeting sialylated epitopes on cancer cells by using pH-activated 5-boronopicolinic acid (5-BPA) as a ligand. **(A)** Design strategy and targeting of overexpressed SA in CSC using pH-sensitive 5-BPA installed polymeric micelles. **(B)** Reduction of CD44+ CSCs in head and neck cancer cells (HSC-2) *in vitro*. **(C)** Improved survival of animal treated with 5-BPA installed polymeric micelles **(D)** Significant reduction in CD44+ CSC population (red) after treatment with 5-BPA installed polymeric micelles. Adapted with permission from reference (88). Copyright 2020 American Chemical Society.

SA is also a promising target for developing targeted therapies. Lectins can target receptors containing sialic acid. For example, a Phase I clinical trial with Maackia amurensis seed lectin (MASL), which can target podoplanin, is set to start for the treatment of squamous cell carcinoma of head and neck (NCT04188665) (163, 164). Podoplanin is a mucin like sialoglycoprotein, co-expressed with CSC marker CD44 and CD44v (165). However, lectins may also bind to healthy cells presenting SA. Moreover, treatment with the SA-cleaving enzyme, neuraminidase, for removing the SA on cancer cells has been explored as a therapeutic approach (166). By conjugating neuraminidase with the HER2-binding antibody trastuzumab (167, 168), it was possible to selectively remove SA from the surface of cancer cells and avoid damaging normal cells. However, systemic injection of such systems could pose major toxicity issues due to the vital role of SA on normal cells and tissues. Thus, it is essential to develop smart ligand-based systems that can only be activated at the tumor microenvironment for specifically targeting SA overexpressed on cancer cells. We have recently reported a novel phenylboronic acid derivative, *i.e.* 5-boronopicolinic acid (5-BPA), with a unique pH-dependent binding profile to SA (169). The binding of 5-BPA to SA and glucose follows opposite trend with reduced pH (169). In other words, 5-BPA molecule favors binding with glucose at pH 7.4, but strongly binds to SA at the acidic tumor microenvironment (pH 7.2-6.5) (170). Thus, 5-BPA can be used to develop pH triggered smart ligand systems, which will only activate in the low pH microenvironment of tumors, avoiding unspecific binding to normal cells. We have recently demonstrated the potential of 5-BPA as a ligand for targeting highly sialylated CD44+ CSCs (**Figure 2A**). Installing 5-BPA molecules on the surface of polymeric micelles loading (1,2-diaminocyclohexane)platinum(II), which is the parent complex of the anticancer drug oxaliplatin, allow reducing CSCs *in vitro* (**Figure 2B**) and also in an orthotopic model of head and neck cancer (**Figure 2D**), improving overall survival (**Figure 2C**) (88). Importantly, as SA is broadly available at the end of glycan chains of different tumors, and tumor acidosis is a hallmark of cancer, 5-BPA could be applied for developing targeted treatments against CSCs in a wide range of tumors.

## Conclusion and Future Perspectives

Here, we briefly presented the effects of glycosylation on CSCs and the possibility to develop therapeutic approaches against such aberrant glycosylation. While glycosylation has been identified to be essential in the signaling pathways and markers of CSC regulating self-renewal, stemness and extravasation, more studies are necessary to unveil differences in glycome and glycosylation of normal cells, stem cells, CSCs and non-CSCs. Such information will allow researchers to develop biomarkers for detecting cancer progression, and precisely target cancer cells and resistant CSCs. Abnormal glycosylation of CSCs plays key role and contributes to their resistance to chemotherapy and ability to metastasize *via* several pathways. While inhibition or manipulation of the glycosylation in CSCs has been shown to be therapeutic, further exploration into the glycosylation associated processes is required to develop effective strategies targeting specific altered markers or signaling pathways without affecting healthy cells. Some approaches, like selectively cleaving surface glycan of tumors, or drugs with affinity for tumor associated glycans, have already demonstrated differential toxicity to tumor cells compared to normal cells, suggesting therapeutic potential through therapeutic window optimization. Moreover, even though CSC markers can present intratumoral and intertumoral heterogeneities, glycosylation could provide relevant targets that are preserved throughout tumors, such as SA, thereby, facilitating the development effective and wide-ranging treatment strategies.

## Author Contributions

TK and HC wrote the manuscript. All authors contributed to the article and approved the submitted version.

## Funding

This work was supported by Grants-in-Aid for Challenging Research (Exploratory) (18K19901; HC) and Grants-in-Aid for Scientific Research B (20H04524 and JP16H03179; HC).

## Conflict of Interest

The authors declare that the research was conducted in the absence of any commercial or financial relationships that could be construed as a potential conflict of interest.
